# Supplementary material for: Natural field diagnosis and molecular confirmation of fungal and bacterial watermelon pathogens in Bangladesh: A case study from the Natore and Sylhet districts
Source: PLoS One. 2024 Nov 7;19(11):e0307245. doi: 10.1371/journal.pone.0307245 (PMC11542843; doi:10.1371/journal.pone.0307245)
Supplement: S1 Appendix — (PDF) [file pone.0307245.s001.pdf]

S1: FASTA nucleotide sequences of the identified fungal and bacterial isolates.

```
1      AAAGTCGTAA AGGTCTCCGT TGGTGAACCA GCGGAGGGAT CATTACTGAG TTTACGCTCT
61     ATAACCCTTT GTGAACATAC CTAACCGTTG CTTCGGCGGG CGGGAGGTCC GCCTCCCCCG
121    GCCCCGCTCG CGGGGAGCCC GCCGGAGGAA AAACCCAACT CTTATGGAAA CGACGTCTCT
181    TCTGAGTGGC ACAAGCAATC AAAACTAACA ACGGATCTCT TGGTTCTGGC ATCGATGAAG
241    AACGCAGCGA AATGATAAGT AATGTGAACA GAATTCAGTG AATCATCGAA TCTTTGAACG
301    CACATTGCGC CCGCCAGCAT TCTGGCGGGC ATGCCTGTTC GAGCGTCATT TCAACCCTAA
361    GCACCGCTTG GCGTTGGGGC TTCCACGGCT GACGTGGGCC CTAAGACAGT GGCGGACCCT
421    CGCGGAGCCT CCTTTGCGTA GTAACATGCC ACCTCGCACC GGGACCCGCA GGGCACTCCT
481    GCCGTAAAAC CCCCCAATTT TTACAAGGTT GACCTCGGAT CAGGTAGGCC GCTGAACTTA
541    AGCA
```

S1-a. Nucleotide sequence of *Colletotrichum orbiculare* isolate NatBD-6, rRNA, partial sequence, (accession no. PP837831).

```
1      TAAGGTTTCG GTAGGTGAAC CTGCGGGAGG GATCATTACT GAGTGAGGGC CTTCGGGCTC
61     GACCTCCAAC CCTTTGTGAA CACAACTTGT TGCTTCGGGG GCGACCCTGC CGTTTCGACG
121    GCGAGCGTTC CCGGAGGCCT TCAAACACTG CATCTTTGCG TCGGAGTTTA AGTAAATTAA
181    ACAAAACTTT CAACAACGGA TCTCTTGGTT CTGGCATCGA TGAAGAACGC AGCGAAATGC
241    GATAAGTAAT GTGAATTGCA GAATTCAGTG AATCATCGAA TCTTTGAACG CACATTGCGC
301    CCCTTGGTAT TCCGAGGGGC ATGCCTGTTC GAGCGTCATT TCACCACTCA AGCCTCGCTT
361    GGTATTCCGC GCCGCGGTGT TCCGCGCGCC TCAAAGTCTC CGGCTGAGCT GTCCGTCTCT
421    AAGCGTTGTG ATTTCATTAA TCGCTTCGGA GCGCGGGCGG TCGCGGCCGT TAAATCTTTC
481    ACAAGGTTGA CCTCGGATCA GGTAGGGATA CCCGCTGAAC TTGTAGCATA TCAATAAAGC
541    CGGAGGAA
```

S1-b. Nucleotide sequence of *Cercospora citrullina* isolate NatBD-7, rRNA, partial sequence, (accession no. PP837832).

```
1     TTCCCTTCGT CCATTTATTT ATATGACACT CAAGCACATA ATCCTTAATT GGCTTCCGGA
61    TCCCACCATG CGCCCATTCT CATGAGGATG TGGTTCCATC TTGAATCGTA GGCGTGCAGG
121   ACAGTAGGCC TCGAGAGTTC CGATAATTTC TTTCCGTAAG GGTAACCTGC GGAGGGATCA
181   TTACCGAGTT TACAACTCCC AAACCCCTGT GAACATACCA CTTGTTGCCT CGGCGGATCA
241   GCCCGCTCCC GGTAAAACGG GACGGCCCGC CAGAGGACCC CTAAAACTCT GTTTCTATAT
301   GTAACTTCTG AGTGGAACCA TAAATAAATC AAAACTTTCA ACAACGGATC TCTTGGTTCT
361   GGCATCGATG AAGAACGCAG CAAAATGCGA TAAGTAATGT GAATTGCAGA ATTCAGTGAA
421   TCATCGAATC TTTGAACGCA CATTGCGAAC GCCAGTATTC TGGCGGGCAT GCCTGTTCGA
481   GCGTCATTTC AACCCTCAAG CACAGCTTGG TGTTGGGACT CGCGTTAATT CGCGTTCCTC
541   AAATTGATTG GCGGTCACGT CGAGCTTCCA TAGCGTAGTA GTAAAACCCT CGTTACTGGT
601   AATCGTCGCG GCCACGCCG TTAAACCCCAA CTTCTGAATG TGACCTCGGA TCATG
```

S1-c. Nucleotide sequence of *Fusarium oxysporum* isolate NatBD-8, rRNA, partial sequence, (accession no. PP837833).

```
1     CCTTCCGTAG GTGAACCTGC GGAAGGATCA TTACCTAGAG TTGCGGGCTT TGCCTGCCAT
61    CTCTTACCCA TGTCTTTTGA GTACCTTCGT TTCCTCGGCG GGTTCGCCCG CCGGTTGGAC
121   AACACTTAAA CCCTTTGTAA TTGAAATCAG CGTCTGTCAA ACTTTAATAG TTACAACTTT
181   CAACAACGGA TCTCTTGGTT CTGGCATCGA TGAAGAACGC AGCGAAATGC GATAAGTAGT
241   GTGAATTGCA GAATTCAGTG AATCATCGAA TCTTTGAACG CACATTGCGC CCCTTGGTAT
301   TCCATGGGGC ATGCCTGTTC GAGCGTCATT TGTACCTTCA AGCTCTGCTT GGTGTTGGGT
361   GTTTGTCTCG CCGCTTGCGC GCAGACTCGC CTCAAAACGA TTGGCAGCCG GCGTATTGAT
421   TTCGGAGCGC AGTACATCTC GCGCTTTGCA CTCACAACGA CGACGTCCAA AAAGTACATT
481   TTACACTCTG ACCTCGGATC AGTAGCTCCC GC
```

S1-d. Nucleotide sequence of *Stagonosporopsis cucurbitacearum* isolate SylBD-1, rRNA, partial sequence, (accession no. PP837834).

```
1     TATAGACCAA AAGGAGAATT ACTGTTCTTG CGGGGTCCAA GTCTTTGCGG TTCTTGGTCA
61    TCTAGAGGAA GTAAAAGTCG TAACAAGGTT TCCGTAGGTG AACCTGCGGA AGGATCATTA
121   CTGAGCGCGA GGCCCCGCAG CGCGCACGCG CTGCGGCGGT TGACCCTCCA CCCGTGTGAA
181   CTCTTATCTG TTGCTTTGGC GGGCCGGGCT CGACCTGCCG GCTCCGGCTG GCGAGTGCCC
241   GTCAGAGAAG CCCCAACTCG TGCTGTGAGT GTTGTATGAG GAAATGTGGA ATTAGTGGAA
301   CTTTTATCAA CGGATCTCTT GGCTCTGGCA TCGATGAAGA ACGCAGCGAA ATGCGATAAG
361   TAATGAGAAT TGCAGAACTT AGTGAATCAT CGAATCCTTG AACGCACATT GCGCCCCCCG
421   GCATTCCGAG GGGCATGCCT GTTCGAGCGT CAGAACCCCC CTCAAGCCTA GCTTGGTCTT
481   GGGGCTCGCC GGCTCGGCGG CCCCTAAACG CAGTGGCGGT GCCGGTGTGC TCTGCCGAGT
541   AGTCATGTAT CTCGCGACAG AGTGGGCGAC GCACCTCACA GAACCCCCAG TTCTATAGGA
601   TGACCTTA
```

S1-e. Nucleotide sequence of *Pseudoperonospora cubensis* isolate SylBD-2, rRNA, partial sequence, (accession no. PP837835).

```
1     CCACACCTAA AAAACTTTCC ACGTGAACCG TATCAACCCT TTAGTTGGGG GTCTTGTACC
61    CTATCATGGC GAATGTTTGG ACTTCGGTCC GGGCGAGTAG CTTTTTGTTT TAAACCCATT
121   TCACAATTCT GATTATACTG TGGGGACGAA AGTCTCTGCT TTTAACTAGA TAGCAACTTT
181   CAGCAGTGGA TGTCTAGGCT CGCACATCGA TGAAGAACGC TGCGAACTGC GATACGTAAT
241   GCGAATTGCA GGATTCAGTG AGTCATCGAA ATTTTGAACG CATATTGCAC TTCCGGGTTA
301   GTCCTGGGAG TATGCCTGTA TCAGTGTCCG TACATCAAAC TTGGCTTTCT TCCTTCCGTG
361   TAGTCGGTGG AGGATGTGCC AGATGTGAAG TGTCTTGCGT GTTGTCCTTC GGGTCGACTG
421   CGAGTCCTTT TAAATGTACT GAACTGTACT TCTCTTTGCT CGAAAAGCGT GGTGTTGCTG
481   GTTGTGGAGG CTGCCTGCGT GGCCAGTCGG CGACCGGGTT GTCTGCTGCG GCGTTTAAAG
541   GAGGAGTGTT CGATTCGCGG TATGGTTGGC TTCGGCTGAA CAGGCGCTTA TTGAATGCTT
601   TTCCTGCTGT GGCGTGATGG GCTGGTGAAC CGTAGCTGTG TTTGGCTTGG CGTTTGAATC
661   GGCTTTGCTG TTGCGAAGTT GAGTGGCGGC TTCGGCTGTC GAGGGTCGAT CCATTTTGGG
721   AACTTTGTGT GCACTTCGGT GCGCATCTCA A
```

S1-f. Nucleotide sequence of *Phytophthora capsici* isolate SylBD-3, rRNA, partial sequence, (accession no. PP837836).

```
1     TCTTCGGATG CTGACGAGTG GCGAACGGGT GAGTAATACA TCGGAACGTG CCCGATCGTG
61    GGGGATAACG AGGCGAAAGC TTTGCTAATA CCGCATAAGA TCTATGGATG AAAGCAGGGG
121   ACCGCAAGGC CTTGCGCGAA CGGAGCGGCC GATGGCAGAT TAGGTAGTTG GTGGGGTAAA
181   GGCTTACCAA GCCTACGATC TGTAGCTGGT CTGAGAGGAC GACCAGCCAC ACTGGGACTG
241   AGACACGGCC CAGACTCCTA CGGGAGGCAG CAGTGGGGAA TTTTGGACAA TGGGCGCAAG
301   CCTGATCCAG CCATGCCGCG TGCAGGATGA AGGCCTTCGG GTTGTAAACT GCTTTTGTAC
361   GGAACGAAAA GCCTTCTTCT AATAAAGGGG GGTCATGACG GTACCGTAAG AATAAGCACC
421   GGCTAACTAC GTGCCAGCAG CCGCGGTAAT ACGTAGGGTG CAAGCGTTAA TCGGAATTAC
481   TGGGCGTAAA GCGTGCGCAG GCGGTGATGT AAGACAGATG TGAAATCCCC GGGCTCAACC
541   TGGGAACTGC ATTTGTGACT GCATCGCTGG AGTACGGCAG AGGGGGATGG AATTCCGCGT
601   GTAGCAGTGA AATGCGTAGA TATGCGGAGG AACACCGATG GCGAAGGCAA TAACCTGGGC
661   CTGTACTGAC GCTCATGCAC GAAAGCGTGG GGAGCAAACA GGATTAGATA CCCTGGTAGT
721   CCACGCCCTA AACGATGTCA ACTGGTTGTT GGGTCTTCAC TGACTCAGTA ACGAAGCTAA
781   CGCGTGAAGT TGACCGCCTG GGGAGTACGG CCGCAAGGTT GAAACTCAAA GGAATTGACG
841   GGGACCCGCA CAAGCGGTGG ATGATGTGGT TTAATTCGAT GCAACGCGAA AAACCTTACC
901   CACCTTTGAC ATGTACGGAA TCCTTTAGAG ATAGAGGAGT GCTCGAAAGA GAACCGTAAC
961   ACAGGTGCTG CATGGCTGTC GTCAGCTCGT GTCGTGAGAT GTTGGGTTAA GTCCCGCAAC
1021  GAGCGCAACC CTTGCCATTA GTTGCTACGA AAGGGCACTC TAATGGGACT GCCGGTGGCA
1081  AACCGGAGGA AGGTGGGGAT GACGTCAAGT CCTCATGGCC CTTATAGGTG GGCTACACA
1141  CGTCATACAA TGGCTGGTAC AGAGGGTTGC CAACCCGCGA GGGGGAGCTA ATCCCATAAA
1201  GCCAGTCGTA GTCCGGATCG CAGTCTGCAA CTCGACTGCG TGAAGTCGGA ATCGCTAGTA
1261  ATCGCGGATC AGCATGTCGC GGTGAATACG TTCCCGGGTC TTGTACACAC CGCCCGTCAC
1321  ACCATGGGAG CGGGTTCTGC CAGAAGTAGG TAGCCTAACC GTAAGGA
```

S1-g. Nucleotide sequence of *Acidovorax citrulli* isolate SylBD-4, 16s rDNA, partial sequence, (accession no. PP837828).

1 CTACACATGC AGTCGAGCGG CAGCACGGGT ACTTGTACCT GGTGGCGAGC GGCGGACGGG
61 TGAGTAATGC CTAGGAATCT GCCTGGTAGT GGGGGATAAC GCTCGGAAAC GGACGCTAAT
121 ACCGCATACG TCCTACGGGA GAAAGCAGGG GACCTTCGGG CCTTGCGCTA TCAGATGAGC
181 CTAGGTCGGA TTAGCTAGTT GGTGAGGTAA TGGCTCACCA AGGCGACGAT CCGTAACTGG
241 TCTGAGAGGA TGATCAGTCA CACTGGAACT GAGACACGGT CCAGACTCCT ACGGGAGGCA
301 GCAGTGGGGA ATATTGGACA ATGGGCGAAA GCCTGATCCA GCCATGCCGC GTGTGTGAAG
361 AAGGTCTTCG GATTGTAAAG CACTTTAAGT TGGGAGGAAG GGCAGTTACC TAATACGTGA
421 TTGTTTTGAC GGTACCGACA GAATAAGCAC CACCTAACTC TGTGCCAGCA GCCGCGGTAA
481 TACAGAGGGT GCAAGCGTTA ATCGGAATTA CTGGGCGTAA AGCGCGCGTA GGTGGTTTGT
541 TAAGTTGAAT GTGAAATCCC CGGGCTCAAC CTGGGAACTG CATCCAAAAC TGGCAAGCTA
601 GAGTATGGTA GAGGGTGGTG GAATTTCCTG TGTAGCGGT GAAATGCGTAG ATATAGGAAG
661 GAACACCAGT GGCGAAGGCG ACCACCTGGA CTGATACTGA CACTGAGGTG CGAAAGGTGG
721 GGAGCAAACA GGATTAGATA CCCTGGTAGT CCACGCCGTA AACGATGTCA ACTAGCCGTT
781 GGGAGCCTTG AGCTCTTAGT GGCGCAGCTA ACGCATTAAG TTGACCGCCT GGGGAGTACG
841 GCCGCAAGGT TAAAACTCAA ATGCCTTGAC GGGGGCCCGC ACAAGCGGTG GAGCATGTGG
901 TTTAATTCGA AGCAACGCGA AGAACCTTAC CAGGCCTTGA CATCCAATGA ACTTTCCAGA
961 GATGGATTGG TGCCTTCGGG AACATTGAGA CAGGTGCTGC ATGGCTGTCG TCAGCTCGTG
1021 TCGTGAGATG TTGGGTTAAG TCCCGTAACG AGCGCAAAAC TTGTCCTTAG TTACCAGCAC
1081 GTTAAGGTGG GCACTCTAAG GAGACTGCCG GTGACAAACC GGAGGAAGGT GGGGATGACG
1141 TCAAGTCATC ATGGCCCTTA CGGCCTGGGC TACACACGTG CTACAATGGT CGGTACAGAG
1201 GGTTGCCAAG CCGCGAGGTG GAGCTAATCT CACAAAACCG ATCGTAGTCC GGATCGCAGT
1261 CTGCAACTCG ACTGCGTGAA GTCGGAATCG CTAGTAATCG CGAATCAGAA TGTCGCGGTG
1321 AATACGTTCC CGGGCCTTGT ACACACCGCC CGTCACACCA TGGGAGTGGG TTGCACCAGA
1381 AGTAGCTAGT CTAACCTTGC GG

S1-h. Nucleotide sequence of *Pseudomonas syringae* isolate SylBD-5, 16s rDNA, partial sequence, (accession no. PP837829).

1	CATGCAGTCG	AGCGGTAGCA	CAGGGGAGCT	TGCTCCCTGG	GTGACGAGCG	GCGGACGGGT
61	GAGTAATGTC	TGGGAAACTG	CCTGATGGAG	GGGGATAACT	ACTGGAAACG	GTAGCTAATA
121	CCGCATAACG	TCGCAAGACC	AAAGAGGGGG	ACCTTCGGGC	CTCTTGCCAT	CAGATGTGCC
181	CAGATGGGAT	TAGCTAGTAG	GTGGGGTAAT	GGCTCACCTA	GGCGACGATC	CCTAGCTGGT
241	CTGAGAGGAT	GACCAGCCAC	ACTGGAACTG	AGACACGGTC	CAGACTCCTA	CGGGAGGCAG
301	CAGTGGGGAA	TATTGCACAA	TGGGCGCAAG	CCTGATGCAG	CCATGCCGCG	TGTGTGAAGA
361	AGGCCTTCGG	GTTGTAAAGC	ACTTTCAGCG	AGGAGGAAGG	TGGTGAGCTT	AATACGCTCA
421	TCAATTGACG	TTACTCGCAG	AAGAAGCACC	GGCTAACTCC	GTGCCAGCAG	CCGCGGTAAT
481	ACGGAGGGTG	CAAGCGTTAA	TCGGAATTAC	TGGGCGTAAA	GCGCACGCAG	GCGGTTTGTT
541	AAGTCAGATG	TGAAATCCCC	GGGCTCAACC	TGGGAACTGC	ATTTGAAACT	GGCAAGCTAG
601	AGTCTCGTAG	AGGGGGGTAG	AATTCCAGGT	GTAGCGGTGA	AATGCGTAGA	GATCTGGAGG
661	AATACCGGTG	GCGAAGGCGG	CCCCCTGGAC	GAAGACTGAC	GCTCAGGTGC	GAAAGCGTCC
721	GGAGCAAACA	GGATTAGATA	CCCTGGTAGT	CCACGCTGTA	AACGATGTCG	ATTTGGAGGT
781	TGTGCCCTTG	AGGCGTGGCT	TCCGGAGCTA	ACGCGTTAAA	TCGACCGCCT	GGGGAGTACG
841	GCCGCCAGGT	TAAAACTCAA	ATGAATTGAC	GGGGGCCCGC	ACAAGCGGTG	GAGCATGTGG
901	TTTAATTCGA	TGCAACGCGA	AGAACCTTAC	CTACTCTTGA	CATCCAGAGA	ACTTAGCAGA
961	GATGCTTTGG	TGCCTTCGGG	AACTCTGAGA	CAGGTGCTGC	ATGGCTGTCG	TCAGCTCGTG
1021	TTGTGAAATG	TTGGGTTAAG	TCCCGCAACG	AGCGCAACCC	TTATCCTTTG	TTGCCAGCGG
1081	TTCGGCCGGG	AACTCAAAGG	AGACTGCCAG	TGATAAACTG	GAGGAAGGTG	GGGATGACGT
1141	CAAGTCATCA	TGGCCCTTAC	GAGTAGGGCT	ACATACGTGC	TACAATGGCA	TATACAAAGA
1201	GAAGCGAGGT	CGCGAGAGCA	AGCGGACCTC	ATAAAGTATG	TCGTAGTCCG	GATTGGAGTC
1261	TGCAACTCGA	CTCCATGAAG	TCGGAATCGC	TAGTAATCGT	AGATCAGAAT	GCTACGGTGA
1321	ATACGTTCCC	GGGCCTTGTA	CACACCGCCC	GTCACACCAT	GGGAGTGGGT	TGCAAAAGAA
1381	GTAGGTAGCT	TAACCTTCGG	GAGGGCGCTA			

S1-i. Nucleotide sequence of *Serratia marcescens* isolate NatBD-9, 16s rDNA, partial sequence, (accession no. PP837830).
