# Supplementary material for: Natural field diagnosis and molecular confirmation of fungal and bacterial watermelon pathogens in Bangladesh: A case study from the Natore and Sylhet districts
Source: PLoS One. 2024 Nov 7;19(11):e0307245. doi: 10.1371/journal.pone.0307245 (PMC11542843; doi:10.1371/journal.pone.0307245)
Supplement: Attachment — Submitted filename: Response to Reviewers.pdf [file pone.0307245.s003.pdf]

Date: 26-09-2024

Academic Editor,
PLOS ONE

Dear Kandasamy Ulaganathan,

The author would like to thank you and the reviewers for your thoughtful and constructive feedback on our manuscript [PONE-D-24-26919]. The author greatly appreciates the time and effort put into reviewing our work, and we have carefully considered each point raised. In response, the author has made the necessary revisions and clarifications throughout the manuscript to address all concerns.

Enclosed is a table detailing each comment made by the academic editor and reviewers, alongside author responses and the corresponding revisions. The author believes these changes have strengthened the manuscript, and we hope that it now meets the journal's standards.

**Academic editor**	**Response by Author**
1. Please ensure that your manuscript meets PLOS ONE's style requirements, including those for file naming.	We have ensured that the manuscript is now fully formatted according to PLOS ONE's style requirements, including file naming conventions. If there are any specific areas that require further adjustment, please let us know, and we will address them accordingly.
2. We suggest you thoroughly copyedit your manuscript for language usage, spelling, and grammar.	We have thoroughly reviewed the manuscript, enhancing the language, spelling, and grammar through careful observation. The text has been edited for clarity, consistency, and readability. If there are any specific sections where further improvements are needed, kindly point them out, and we will address them immediately.
3. Your ethics statement should only appear in the Methods section of your manuscript.	The ethics statement has been moved to the Methods section as per the journal's guidelines.
4. Please include captions for your Supporting Information files at the end of your manuscript, and update any in-text citations to match accordingly.	Captions for the Supporting Information files have been added at the end of the manuscript, and all in-text citations have been updated to match accordingly.
5. PLOS ONE now requires that authors provide the original uncropped and unadjusted images underlying all blot or gel results	We have ensured that all figures adhere to PLOS ONE's guidelines. The original, uncropped, and unadjusted images underlying all blot or gel data reported in the manuscript have been provided as required.

reported in a submission's figures or Supporting Information files.	
**Reviewer #1**	
1. The study lacks novelty and is of local importance.	We appreciate the reviewer's feedback regarding the novelty and local importance of the study. While we acknowledge that the study focuses on a specific region (Bangladesh), it addresses a critical issue from a quarantine and agricultural export perspective. Identifying and documenting potential diseases in watermelon is crucial for the Bangladeshi government and regulatory authorities to ensure food safety, prevent disease outbreaks, and protect local agriculture.<br>Furthermore, this research holds international relevance, as it provides critical insights to global stakeholders, particularly those involved in importing watermelon from Bangladesh. By identifying any new or emerging watermelon diseases in the region, we offer valuable information that can help importing countries implement appropriate quarantine measures and protect their agricultural sectors from potential threats. Therefore, while rooted in a local context, the findings have wider implications for international trade, biosecurity, and disease management.<br>We hope this clarification emphasizes the significance of the study beyond its local scope and provides a better understanding of its broader impact.
**Response to Reviewer #2**	
1. Keywords section, remove one keyword	We appreciate the reviewer's suggestion. The keyword "PCR" has been removed from the Keywords section as requested.
2. In the introduction section, add one paragraph with the latest citation.	We appreciate the reviewer's suggestion to add a paragraph with the latest citation. Upon reviewing the Introduction section, we believe that all current citations are both relevant and up-to-date. However, if there are specific points or topics you feel should be further elaborated upon, we would welcome your suggestions to ensure the introduction fully addresses all relevant aspects of the study.<br>Additionally, if you have identified any citations that may no longer reflect the most recent findings, we would be happy to replace them with more current references upon your recommendation. We value your input and are committed to ensuring the highest quality and accuracy in our work.
3. Add the reference PMID: 35867657	We appreciate the reviewer's suggestion to add the reference PMID: 35867657. However, upon reviewing the paper titled "**Structural insights into the RNA interaction with Yam bean Mosaic virus (coat protein) from *Pachyrhizus erosus* using bioinformatics approach,"** we found that this study focuses on RNA interaction with viral diseases, which is not directly relevant to our research focus.

4. Add few Reference in the reference section of the manuscript.	Thank you for your suggestion to add additional references in the reference section. Currently, there are 27 references included in the reference section, all of which have been cited within the manuscript.<br>If you have identified any citations that are missing from the reference list or if there are specific references that you believe should be added to strengthen the manuscript, kindly indicate the corresponding reference number(s). We would be happy to make the necessary additions or corrections based on your feedback
5. Discuss more in the Discussion section of the manuscript with citations.	We have added additional essential points to the Discussion section, along with relevant citations, to provide a more comprehensive analysis of the study's findings. These new points address the emerging threats posed by Fusarium wilt and Gummy stem blight in watermelon production and their potential impact, referencing recent studies that support our observations. We hope this expanded discussion meets the reviewer's expectations.
6. If the author provide the samples location then it will be better for the study.	The sample locations, including block areas and Upazila names, have been provided in **Table 1.** Additionally, the table includes relevant information on these locations' climatic and soil conditions to give a more comprehensive context for the study. We hope this adequately addresses your concern and enhances the clarity of the study.
7. Describe the details of the sample collection methods	The sample collection methods have now been described in detail in the revised manuscript. The updated section provides comprehensive information on the process, including how symptomatic plant parts were selected, stored, labeled, and transported, as well as the steps taken to ensure the integrity of the samples during collection and laboratory analysis. We hope this addresses your concerns and enhances the clarity of our methodology.
8. Why the author use Maximum Likelihood method? why not NJ method?	We appreciate the reviewer's inquiry regarding the use of the Maximum Likelihood (ML) method over the Neighbor-Joining (NJ) method in our analysis. The decision to use the ML method was based on its advantages in providing more robust and accurate phylogenetic estimations.<br>The NJ method, while faster and computationally less demanding, is a distance-based approach and does not account for the probabilistic nature of evolutionary changes, which can lead to less precise results, particularly for datasets with high variability. For our study, the higher precision and reliability offered by the ML method were essential for producing accurate phylogenetic inferences, which is why it was chosen for the MEGA analysis.
9. Check the grammatical mistake throughout the manuscript.	Thank you for your observation. We have thoroughly reviewed the manuscript multiple times and addressed any grammatical

	issues that were identified. If there are specific sections where you notice errors or believe further improvements are needed, kindly point them out, and we will make the necessary revisions. We are committed to ensuring the manuscript is grammatically sound.

We look forward to your further evaluation of our revised manuscript.

Sincerely,

Raihan Ferdous

Corresponding author
